# Good practices and challenges in addressing poliomyelitis and measles in the European Union

**DOI:** 10.1093/eurpub/cky056

**Published:** 2018-04-06

**Authors:** John Kinsman, Svenja Stöven, Fredrik Elgh, Pilar Murillo, Michael Sulzner

**Affiliations:** 1 Epidemiology and Global Health Unit, Department of Public Health and Clinical Medicine, Umeå University, Umeå, Sweden; 2 European CBRNE Centre, Umeå University, Umeå, Sweden; 3 Department of Clinical Microbiology, Umeå University, Umeå, Sweden; 4 Crisis Management and Preparedness in Health Unit, Public Health Directorate, Directorate-General for Health and Food Safety (DG SANTE), European Commission, Luxembourg, Luxembourg

## Abstract

**Background:**

All European Union (EU) and European Economic Area (EEA) Member States have pledged to ensure political commitment towards sustaining the region’s poliomyelitis-free status and eliminating measles. However, there remain significant gaps between policy and practice in many countries. This article reports on an assessment conducted for the European Commission that aimed to support improvements in preparedness and response to poliomyelitis and measles in Europe.

**Methods:**

A documentary review was complemented by qualitative interviews with professionals working in International and EU agencies, and in at-risk or recently affected EU/EEA Member States (six each for poliomyelitis and measles). Twenty-six interviews were conducted on poliomyelitis and 24 on measles; the data were subjected to thematic analysis. Preliminary findings were then discussed at a Consensus Workshop with 22 of the interviewees and eight other experts.

**Results:**

Generic or disease-specific plans exist in the participating countries and cross-border communications during outbreaks were generally reported as satisfactory. However, surveillance systems are of uneven quality, and clinical expertise for the two diseases is limited by a lack of experience. Serious breaches of protocol have recently been reported from companies producing poliomyelitis vaccines, and vaccine coverage rates for both diseases were also sub-optimal. A set of suggested good practices to address these and other challenges is presented.

**Conclusions:**

Poliomyelitis and measles should be brought fully onto the policy agendas of all EU/EEA Member States, and adequate resources provided to address them. Each country must abide by the relevant commitments that they have already made.

## Introduction

European Union (EU) Decision 1082/2013/EU (October 2013) on serious cross-border health threats sets out the legal basis for collaboration and information exchange between Member States of the EU, as well as between international and European level institutions on preparedness, prevention and mitigation in the event of a public health emergency.^1^ Among the diseases that have emerged as being of particular importance within the context of EU Decision 1082 are poliomyelitis and measles.^2^ All EU and European Economic Area (EEA) Member States have pledged to ensure political commitment towards (i) sustaining the region’s poliomyelitis-free status and (ii) eliminating measles, which constitute two of the six goals of the WHO’s European Vaccine Action Plan.^3^

There are, however, still areas that require attention to achieve the European goals related to these two vaccine-preventable diseases. With regard to poliomyelitis, in spite of the considerable overall historical success of the Global Poliomyelitis Eradication Initiative, ongoing transmission of poliomyelitis is currently considered to be a Public Health Emergency of International Concern,^4^ with particular concerns raised in 2016 about Pakistan, Afghanistan and Nigeria.^5^ Europe has been poliomyelitis-free since 2002^6^ but eight EU/EEA countries were designated in 2015 by the WHO Regional Certification Commission for Poliomyelitis Eradication as being at either ‘intermediate’ or ‘high’ risk of importation and subsequent transmission of poliomyelitis;^7^ and 11 EU/EEA countries were designated as such in 2016.^8^ The approach to sustaining the region’s poliomyelitis-free status is defined by the Poliomyelitis Eradication and Endgame Strategic Plan, which, within Europe, requires a particular focus on poliomyelitis containment alongside continuous high vaccination coverage.^9^

With regard to measles, WHO calls for at least 95% coverage with both the first and second routine doses of measles vaccine (or measles–rubella-containing vaccine, as appropriate) in each district and nationally, for every country in the world.^10^ The European Centre for Disease Prevention and Control (ECDC) reported that, in 2014, 16 of the 31 EU/EEA countries were above the coverage target of 95% for the first dose and just six countries were above this target for the second dose, indicating a significant gap that needs to be filled.^11^ In addition, 21% of the measles cases in 2015 for whom age was known in the EU/EEA were aged 15 years and older,^12^ suggesting that catch-up campaigns with teenagers and adults are needed to close immunization gaps in older populations, alongside improvements in routine vaccination coverage for younger children.

This article reports on an assessment conducted for the European Commission in 2016 that aimed to identify strengths and opportunities for EU/EEA Member States, international institutions and EU agencies to improve preparedness and response to poliomyelitis and measles.

## Methods

Two complementary types of data were collected for this multi-level, qualitative study: documentation and interviews. Material for the documentary review was derived from government, EU and WHO sources, supplemented as appropriate by articles in the peer-reviewed literature. Since our focus was specifically on the policy and technical level authorities who are providing the services as opposed to the target populations, interviews were sought with professionals working in (i) International agencies such as WHO (Geneva and Copenhagen), the International Office for Migration and United Nations Children's Fund (UNICEF); (ii) The European Commission and ECDC and (iii) EU/EEA Member States. For poliomyelitis, the Member States comprised the eight countries with an ‘intermediate’ or ‘high’-risk score for importation and subsequent transmission of poliomyelitis virus as described in the 2015 report of the WHO Europe Regional Certification Committee;^7^ for measles, they comprised the seven countries with an annual measles notification rate, as reported by ECDC, of >5 per million population between October 2014 and September 2015.^11^

From each participating country, we aimed to conduct interviews with four people, two each from the health and relevant non-health sectors. Respondent categories included state epidemiologists, national vaccine managers, surveillance coordinators, health officers in the Ministries of Education and the Interior, and health journalists. Interviewees were identified either through the European Commission, through professional acquaintance or by following up names identified in the literature. In total, we conducted 26 interviews for poliomyelitis (10 from the international and EU levels and 16 from six EU/EEA Member States) and 24 interviews for measles (six from the international/EU levels and 18 from six EU Member States). Note that some interviewees at the international and EU levels answered questions about both poliomyelitis and measles. See table 1.

**Table 1:** The numbers of interviews conducted for poliomyelitis and measles at International, EU and national levels; and the names of the countries included for the national level interviews

	International/EU level	National level (names of countries)	Total number of interviews
Poliomyelitis	10	16 (Bulgaria, Cyprus, Iceland, Latvia, Norway and Romania)	26
Measles	6	18 (Austria, Belgium, Croatia, France, Germany and Lithuania)	24

The interviews were conducted between April and June 2016, mostly over the phone, but, with some of the international level respondents, face-to-face at their offices in Geneva and Copenhagen. The questions were sent in advance to each interviewee to give them the opportunity to consider and prepare their answers. The focus was on actual experiences from preparedness and response activities against poliomyelitis and measles; challenges encountered during these activities, and the means by which these challenges were addressed. Interviewees also received an information sheet about the study which stressed that their participation was voluntary, that they would be taking part purely on the basis of their professional experience and knowledge, and that confidentiality and anonymity would be guaranteed. See the Supplementary data. All the interviews were conducted by one senior expert, with extensive notes of the conversations taken by another team member. These notes constituted the data used in the analysis.

The data were subjected to thematic analysis, with the aim of highlighting lessons learned as well as gaps and challenges. The issues that emerged through this process were discussed and further developed at a Consensus Workshop held in Luxembourg in September 2016, with 22 of the interviewees in attendance alongside eight other invited experts. The suggestions and points made in this article, which has also been presented in expanded form as a report for the European Commission, are the outcome of the interviews and subsequent workshop discussions.

## Results

### Poliomyelitis-specific issues

No major problems were anticipated by the national level interviewees in the event of a poliomyelitis event or outbreak in their countries. Although individual cases may conceivably emerge due to travel from endemic areas, it was felt that the chance of a sustained outbreak is very low: they were confident that the system would work and that funds and vaccines would be available as needed.

Although WHO recommends that countries have a poliomyelitis-specific plan, it was seen as one of several diseases, within a European context, for which a generic plan would suffice. As long as the plan is flexible, and as long as roles and responsibilities for the different actors and sectors involved are sketched out for activities related to mass vaccination, surveillance and containment, a generic plan for vaccine-preventable diseases was seen as sufficient and more cost-effective than a poliomyelitis-specific plan.

Acute Flaccid Paralysis surveillance is recognized globally as a mainstay of poliomyelitis surveillance,^13^ and while five of the six participating countries have adopted it, there were questions about its reliability. Most doctors in Europe never have seen a case of acute poliomyelitis, so ongoing training is needed to maintain the levels of knowledge required to make a correct diagnosis. Environmental surveillance^13^ was seen, for Europe, as a more sensitive and cost-effective method capable of confirming the poliomyelitis-free status of a country and of detecting circulating virus. However, we were told that care needs to be taken to align sampling schemes with population density and mobility.

A major focus for all countries should be on poliovirus containment and on meeting the objectives of GAP III (the current version of the Global Action Plan on poliomyelitis containment).^14^ All countries have committed through Resolution 68.3 of the World Health Assembly,^15^ GAP III itself^14^ and EU Directive 2000/54/EC^16^ to produce inventories of past and present storage sites for materials that may potentially contain poliovirus, and to destroy material that is not considered essential. We found, however, that several countries did not appear to be fully committed to these vital international agreements, and serious breaches of protocol have also recently been reported from companies producing poliomyelitis vaccines.^4^^,^^17^ It was suggested that a central authority in each of these countries should be given legal power as well as sufficient resources to supervise the inventory and to enforce containment procedures.

All European countries currently use inactivated poliomyelitis vaccine (IPV) in their vaccination programmes, which is highly effective against paralysis, but which does not prevent virus multiplication inside the intestine, potentially thereby facilitating continued circulation in the population. By contrast, oral poliomyelitis vaccine (OPV) does induce strong intestinal immunity and it is therefore used in outbreak situations; however, it is not as safe as IPV^9^ and it is not legally permitted in some countries. It was not clear from the interviews how emergency mass vaccination would be facilitated in countries where the legal framework prevents the use of OPV even in the event of an outbreak. We were advised that countries should review their legal framework and their arrangements for the implementation of mass vaccination so that immunization with OPV would be permitted.

The primary issue of concern for poliomyelitis was to ensure high levels of population immunity. There was consensus that the reason for insufficient vaccine coverage in many countries was, ultimately, inadequate political commitment at national level.

There were strong calls from our respondents for their governments to abide by all the agreements concerning both poliomyelitis vaccination and containment which they have signed—including Resolution 68.3 of the World Health Assembly,^15^ WHO’s European Vaccine Action Plan,^3^ the Poliomyelitis Eradication Endgame Strategic Plan^9^ and the Conclusions of the EU on the role of vaccination for public health.^18^

### Measles-specific issues

By contrast with poliomyelitis, most of the participating countries reported high levels of national level political will to eliminate measles, as per WHO’s European Vaccine Action Plan (to which, as indicated earlier, they are all signatories).^3^ However, national commitment was not translated evenly into regional and local implementation. Factors such as political, financial and operational structures shaped regional responses, as evidenced by differences in the incidence of measles in various regions of these countries.^11^

National and local measles plans exist in a majority of the countries. These include strategic plans, as well as procedures for vaccination campaigns, the provision of public information about measles, and conducting outreach missions to at-risk communities such as migrants and Roma people. Plans differed substantially between countries, however, and several did not cover the full spectrum of tasks needed for effective vaccination coverage and outbreak control. Further, plans were not always easy to access and they are not always regularly updated. An additional, major concern is that although plans exist for some countries, it is not always possible to fully implement them due to financial and/or personnel constraints.

Delays in response were observed at the start of some outbreaks due to a combination of: delayed diagnosis, caused by low clinical awareness of measles; inefficiencies in notification systems that are paper-based rather than computer-based; and insufficient laboratory capacity. These collectively allowed outbreaks to spread rapidly before effective control measures were put in place. Key areas that were highlighted as needing additional focus included (i) improving knowledge about the disease and vaccination, both for the public and for health workers; (ii) the development and institution of an electronic notification system connected to all levels of the health system, to which health workers are obliged to notify suspected measles cases within 24 h; and (iii) standard operating procedures for measles with regard to contact tracing, ring vaccination and post-exposure prophylaxis.

Although the administration of two measles vaccine doses is universally required by national vaccination programmes, measles vaccination is not mandatory in all the participating countries. Where vaccinations are covered by health insurance, not everyone is covered, so children may go unvaccinated because of parents’ financial constraints. Many adults are also not protected from measles. Low measles vaccine coverage (especially for the second dose) was ascribed in many cases to health systems issues, including a failure to inform parents of the need to vaccinate their children, as well as a failure to provide infrastructural support to health workers to provide vaccinations. We were informed that policy makers need to prioritize the provision of adequate incentives and infrastructural support to health workers to ensure that measles vaccine coverage rates are optimized.

### Cross-cutting issues

The 2008 economic crisis had a great impact on health systems in many European countries, including on their capacity to implement vaccination programmes and to support polio containment activities. However, reductions in funding and staffing levels were seen as due not only to the economic crisis but, in at least some cases, also to a lack of meaningful political commitment to allocate adequate financial resources to the public health system.

In general terms, and in spite of language difficulties in some cases, cross-border communication between EU Member States was said to be good. Within the EU, the Early Warning and Response System^19^ and the Epidemic Intelligence Information System^20^ provide easily utilized and effective means of communicating with neighbouring Member States in the event of an outbreak. It was suggested that mechanisms to complement the International Health Regulations^21^ could be developed to enhance and improve communications with neighbours who are not EU Member States.

Efforts to improve vaccine coverage rates should, we were informed, focus both on the health system and on the community. Practices that were proposed as having potential to improve vaccine coverage rates included the following (note that these are not presented in any order of perceived importance):

#### Promoting access

1. Ensuring that vaccines and vaccination services are easily accessible, such as through user-friendly, ‘one-stop shops’.
2. Enacting legislation to ensure that vaccination is free of charge to all, including asylum seekers, migrants, refugees, people who are uninsured, and potentially high-risk employees (health workers, migration, police, border control, etc.).
3. Using trained health mediators to work with Roma and other under-served communities.

#### Schools

1. Inclusion of vaccination as a topic in the school curriculum, thereby bringing about long-term benefits in coverage rates by reaching out to future parents.
2. Requiring parents to provide schools with documentation to show that they have been informed about vaccination recommendations for their children.
3. Reviewing children’s vaccination status at school on a regular basis, as a means of identifying at-risk children and encouraging their parents to have them vaccinated.
4. Enacting legislation to prohibit unvaccinated children from attending school during a measles outbreak.
5. Use of smartphone apps that send parents reminders about their children’s vaccinations.

#### Health workers

1. Supporting health workers, including through training and resources, to act as effective risk communicators and vaccination advocates at community level.
2. Guaranteeing that health workers have legal protection in the event that a patient suffers from adverse effects following vaccination.

#### Research

1. Making systematic efforts to understand the reasons for low vaccination uptake rates through WHO-EURO’s *Tailoring Immunization Programmes*,^22^ and acting on the findings.
2. Using vaccination coverage surveys to identify potential geographical and demographic immunity gaps, thereby providing a strong evidence base for where catch-up vaccination programmes may be needed.
3. Evaluating vaccination campaigns as a means of maximizing effectiveness and cost-effectiveness in future.

#### Official websites

1. Significantly improving the standard of many official public health websites, and ensuring that they are kept up to date. Criteria for quality public health websites are available at WHO’s Vaccine Safety Net project.^23^
2. Making serious efforts to bring these high quality official public health websites to the top of internet search lists, as a counter to anti-vaccine websites.
3. Including frequently asked questions on official public health websites, with responses to issues about vaccination that are observed to be circulating online.
4. Using pre-existing material from ECDC [‘Let’s talk about hesitancy’^24^ and ‘Let’s talk about protection’^25^] and from WHO-EURO [‘How to respond to concerns about vaccination’^26^] as recognized means of countering vaccine hesitancy.

#### Political and public health authorities

1. Ensuring that official spokespeople during outbreaks are media-trained and trusted by the public.
2. Targeting political leaders with comprehensive and correct information about vaccination so that they do not disseminate messages that undermine vaccination campaigns.

## Discussion

The methodology adopted for this study into good practices and challenges faced in addressing poliomyelitis and measles in the EU/EEA has facilitated an unusually broad analysis of current thinking about these two vaccine-preventable diseases. The findings presented in this article can be seen as reflecting the majority of the core issues that technical experts face across Europe, as well as a range of good practices that they themselves have identified as means of addressing the various challenges inherent in their work.

In general terms, we found that poliomyelitis was not prioritized by national level decision makers as much as measles, perhaps because Europe has now been polio-free for 15 years, and it simply is not seen as a major problem. In spite of this difference, however, unacceptable immunity gaps remain the norm for both measles and poliomyelitis in many populations.^7^^,^^11^ Further, lax safety standards by poliomyelitis vaccine producers have recently led to dangerous releases of live poliovirus.^4^^,^^17^ Together, these points suggest that in spite of the high-level agreements made by all EU/EEA Member States to prioritize poliomyelitis and measles control,^1^^,^^3^^,^^9^^,^^14–16^^,^^18^ there remains a significant gap between vaccination and containment policy and practice in settings across Europe.

New legislation at European level to bring about effective poliomyelitis containment and to close existing immunity gaps in the population for both measles and poliomyelitis was not seen by our respondents as either necessary or viable. Rather, the focus now should be on ensuring that poliomyelitis and measles are brought fully onto the policy agendas of all EU/EEA Member States, and that each country abides by the relevant commitments that they have already made.

## Supplementary data

Supplementary data are available at *EURPUB* online.

## Supplementary Material

Supplementary Data
