# Supplementary material for: Good practices and challenges in addressing poliomyelitis and measles in the European Union
Source: Eur J Public Health. 2018 Apr 6;28(4):730–4. doi: 10.1093/eurpub/cky056 (PMC6051453; doi:10.1093/eurpub/cky056)
Supplement: Supplementary Data [file cky056_ejph-2017-11-om-0927-file002.pdf]

Supplementary File

Good practices and challenges in addressing poliomyelitis and measles in the European Union (John Kinsman, Svenja Stöven, Fredrik Elgh, Pilar Murillo, Michael Sulzner)

1. Interview information sheet – EU and International Agencies
2. Interview information sheet – EU Member States
3. Measles and polio questions – EU and International Agencies
4. Measles questions – EU Member States
5. Polio questions – EU Member States

Assessments of good practices in addressing selected health threats in the EU/EEA
Specific Contract No. 2015 72 02

Interviewee Information Sheet – EU and International Agencies

The European Commission would like to request your participation in an Assessment of good practices in addressing three diseases – Middle East Respiratory Syndrome (MERS), Measles, and Poliomyelitis – in the EU/EEA. Eighteen countries are included in this Assessment, on the basis of their having had at least one MERS case; a measles notification rate during the 12 months from October 2014-September 2015 of >5 per million population; or their having received a Composite Risk Score of 'Intermediate' or 'High' in the 2015 European Regional Certification Commission for Poliomyelitis Eradication annual report. A number of international and EU agencies and institutions with involvement in one or more of these diseases are also included.

The legal framework and contractor for the Assessment

This Assessment is organized under the Health Programme (2014-2020) within the frame of Specific Contract 2015 72 02, signed between the CELESTE consortium and the Consumers, Health, Agriculture and Food executive Agency (Chafea), acting on a mandate from the European Commission. The CELESTE consortium consists of four partners: Public Health England; the Swedish Defence Research Agency; Umeå University (in Sweden); and Istituto Superiore di Sanita (in Italy). The Assessment will be carried out by the Umeå University consortium partner, with support from Public Health England, by experts in risk and crisis communication, communicable diseases, and preparedness and response to public health threats.

Objective of the Assessment

The overall objective of the Assessment is to identify strengths and opportunities for Member States, international institutions, and EU agencies to improve preparedness to respond to serious cross border threats to health. The Assessments are taking place within the context of EU Decision 1082/2013/EU, which highlights the importance of inter-sectorial and cross-border collaboration in responding to such threats. The Assessment will be conducted through documentary review and interviews (in English).

Your proposed role in the Assessment

You have been contacted to contribute to this Assessment because of your position in [ORGANISATION], and because of the critical role that you and [ORGANISATION] play in global preparedness and response to cross-border health threats.

Your participation in this Assessment is of course voluntary, but if you agree to take part, we would like to interview you in your professional capacity in relation to your experiences and

knowledge of the three diseases in question, specifically from a European perspective. We will take extensive notes during the interview; the information you provide us with will be put together with interview and documentary material from various countries across Europe as well as international and EU institutions and agencies, and will thereby contribute to a series of reports that we will produce concerning good practices and lessons learned. These reports may subsequently be developed further for publication in the peer-reviewed academic literature. We guarantee that you and your contribution will remain anonymous in any and all presentations and publications coming out of this work.

Output of the Assessment

We will analyse the material collected from the 18 participating countries and the international and EU institutions and agencies, and present the draft findings for the three diseases at a Consensus Workshop to be held in Luxembourg in September. We expect up to two people from each of the 18 participating countries, and we will also invite participants from other EU/EEA countries and relevant international and EU institutions and agencies. Delegates will be expected to provide critique and input into the draft findings, based on which the findings will be finalised and presented at a Dissemination meeting in Luxembourg in December. The Dissemination meeting will target policy makers.

We very much hope that you will be interested in contributing to this important Assessment. We believe it will provide a unique opportunity for the exchange of lessons learned, and lead to greater health security across Europe.

Please contact Associate Professor John Kinsman (john.kinsman@umu.se), who will be conducting the interviews, if you have any questions about the content of the work.

Assessments of good practices in addressing selected health threats in the EU/EEA
Specific Contract No. 2015 72 02

<u>Interviewee Information Sheet – EU Member States</u>

The European Commission would like to request your participation in an Assessment of good practices in addressing three diseases – Middle East Respiratory Syndrome (MERS), Measles, and Poliomyelitis – in the EU/EEA. Croatia is one of 18 countries included in this Assessment, on the basis of the measles epidemic that struck the country during the first half of 2015.

The legal framework and contractor for the Assessment

This Assessment is organized under the Health Programme (2014-2020) within the frame of Specific Contract 2015 72 02, signed between the CELESTE consortium and the Consumers, Health, Agriculture and Food executive Agency (Chafea), acting on a mandate from the European Commission. The CELESTE consortium consists of four partners: Public Health England; the Swedish Defence Research Agency; Umeå University (in Sweden); and Istituto Superiore di Sanita (in Italy). The Assessment will be carried out by the Umeå University consortium partner, with support from Public Health England, by experts in risk and crisis communication, communicable diseases, and preparedness and response to public health threats.

Objective of the Assessment

The overall objective of the Assessment is to identify strengths and opportunities for Member States, international institutions, and EU agencies to improve preparedness to respond to serious cross border threats to health. The Assessments are taking place within the context of EU Decision 1082/2013/EU, which highlights the importance of inter-sectorial and cross-border collaboration in responding to such threats. The Assessment will be conducted through documentary review and phone interview (in English).

Your proposed role in the Assessment

You have been contacted to contribute to this Assessment because you fall into one of four different categories of professional who are being interviewed by phone in each participating country about measles/polio [delete as appropriate]: **two** from the health sector (the State epidemiologist, and either someone from the National immunization programme or someone from the National paediatrician organization), and **two** from relevant non-health sectors (someone engaged in education who has knowledge of measles issues in schools, and a health journalist from the national media).

Your participation in this Assessment is of course voluntary, but if you agree to take part, we would like to interview you by phone in your professional capacity in relation to your experiences and knowledge of the measles epidemic. We will take extensive notes during the

interview; the information you provide us with will be put together with interview and documentary material from your own country and from other countries across Europe, and will thereby contribute to a series of reports that we will produce concerning measles good practices and lessons learned. These reports will be shared with other EU/EAA Member States, and they may subsequently be developed further for publications in the peer-reviewed academic literature. We guarantee that you and your contribution will remain anonymous in any and all presentations and publications coming out of this work.

Output of the Assessment

The contractors will analyse the material collected from the 18 participating countries, and present the draft findings for the three diseases at a Consensus Workshop to be held in Luxembourg in September. We expect up to two people from each of the 18 participating countries to attend and we will also invite participants from other EU/EEA countries and relevant international and EU institutions and agencies. Delegates will be expected to provide critique and input into the draft findings, based on which the findings will be finalised and presented at a Dissemination meeting in Luxembourg in December. The Dissemination meeting will target policy makers.

We very much hope that you will be interested in contributing to this important Assessment. We believe it will provide a unique opportunity for the exchange of lessons learned, and lead to greater health security across Europe.

Please contact Associate Professor John Kinsman, Team Leader (john.kinsman@umu.se), and Professor Fredrik Elgh, who will be conducting the interviews (fredrik.elgh@umu.se) if you have any questions about the content of the work; or Lia Olsson (lia.olsson@umu.se) regarding the organisation and timing of your interview.

Measles and polio questions – EU and International Agencies

Institutional issues; and preparedness and response activities and plans

1. How would you describe [ORGANISATION] institutional role in preparedness and response to polio and measles in Europe? Which other institutions (i.e. in addition to Member States) do you engage with on these issues, and how?
2. What does Accelerated Disease Control involve? How is it operationalized across Europe?
3. How useful and/or effective do you think EU Decision 1082 is in terms of facilitating polio and measles preparedness and response activities in Europe? What could be improved to make it more effective? Which do you think are the most important non-health sectors in these activities?
4. In general, how do countries address the situation when they receive an 'Intermediate' or 'High' risk assignation from the RCC; or 'endemic' measles status from the RVC? Do they usually act effectively, or do some not manage? For those that do not, what are the main reasons, and what can be done to improve the situation?
5. What steps are taken, by [ORGANISATION] or other agencies, to help smaller countries (where the reporting burden is proportionately larger) complete their annual reports to the RCC and RVC, and establish the relevant national commissions etc (e.g. Monaco and San Marino for measles)?
6. Do you consider that the three officially sanctioned reporting and notification systems (IHR, EWRS, TESSy) provide the authorities at the European level with adequate polio- and measles-relevant information? To what extent is there overlap in these systems? What are the main challenges in the European VPD reporting and notification efforts, if any? What, if anything, could be improved?
7. Do you think that, in general, sufficient steps are taken by both the international/EU agencies and Member States across Europe (a) to promote polio and measles vaccination, and (b) to address vaccine hesitancy?

Polio

8. In relation to Europe, are you optimistic about the Polio Eradication and Endgame Strategic Plan 2013-2018? Where do you see the biggest challenges in operationalizing this plan? Would you say there is good political commitment across Europe to implement it? If not, why not, and what could be done to improve the commitment?
9. How, if at all, do you think that Europe's polio-free status since 2002 has affected polio preparedness planning (and awareness) in countries across Europe?
10. Do you think that the steps taken by both the international/EU agencies and Member States to address the 2013 Syrian polio cases and the 2015 cVDPV Ukraine cases were sufficient? Was there anything more that should or could have been done? If so, why were these actions not taken? What have been the main lessons learned from these experiences?

11. In the post-tOPV era, what do you see as the main challenges for polio prevention activities in Europe? How can these challenges be addressed?

Measles

12. What would you say were the main lessons learned from the Berlin and Alsace measles epidemics of 2014-2015?
13. What do you think will be the impact (politically and operationally), if any, of the failure to reach the 2015 measles elimination target for Europe?
14. What do you consider to be the main challenges to eliminating measles from Europe, and what can be done to address these challenges?

General

15. Are there any key documents related to these issues that you can recommend for us?
16. Are you satisfied with the arrangements (financial, logistical, security) at European level for storage, transport and analysis of suspected polio samples?
17. What would you say are the key health implications associated with the influx of refugees coming into Europe at the moment, and what practical steps should be taken to reduce any risks? Are these steps being taken, and if not, why not?
18. In what ways does the European Vaccine Action Plan help to maintain Europe's polio-free status?
19. Are you satisfied with the accuracy of routine polio and measles immunization coverage data in European countries, both for the whole population and for any under-vaccinated groups?

MEASLES QUESTIONS – EU Member States

	Health sector		Non-health sector	
	**State epidemiologist**	**National immunization programme/ National paediatrician organisation**	**Education Ministry**	**Health journalist**
**GENERAL**				
1. Describe the actions that were taken in response to the recent measles epidemic in your country. Which were the key institutional actors, and which non-health-related sectors, if any, were involved?	**X**	**X**		
2. What were the main challenges faced during the response to the recent measles epidemic, and how were these addressed?	**X**	**X**		
3. Does a written national measles control plan exist, or was the response based on a generic plan? If there is a plan, when was it last updated? Is this document publicly available? [See if we can get a copy]	**X**			
4. What was your institutional role in responding to the recent measles epidemic? Is this role defined in a formal protocol?	**X**	**X**	**X**	
5. What, if anything, do you think could have been done to improve your institution's response to the recent measles epidemic?	**X**	**X**	**X**	
6. To what extent did organizational issues in the health system affect the response to the recent measles epidemic? Details.	**X**	**X**		
7. What funding mechanisms are in place to ensure that measles response activities are properly supported? Were there any challenges in this during the recent measles epidemic? Details.	**X**	**X**		

MEASLES QUESTIONS – EU Member States

VACCINATION AND HEALTH SYSTEMS ISSUES				
8. Are you satisfied by the accuracy of routine measles immunization coverage data in the country, both for the whole population and for any hard-to-reach groups? Please explain.		**X**		
9. Can you describe the system for measles vaccination in the country?		**X**		
10. What are the first and second dose measles vaccination coverage rates in the country? How have these fluctuated over recent years? What are the challenges in achieving the 95% coverage rate required for population immunity?		**X**		
11. What steps are currently taken to promote routine measles vaccination?		**X**		
12. What is known about community perceptions and attitudes towards vaccination? Was this knowledge used to promote vaccination during the recent measles epidemic? Details.	**X**	**X**		
13. To what extent does vaccine hesitancy exist in the country? Which groups are most resistant to vaccination, and what is it that drives their resistance?	**X**	**X**		
14. Is there currently a strategy for increasing measles vaccination coverage (i) in the general population and (ii) for refugee and lower-uptake populations? What are the challenges in this, and how are these challenges being addressed?		**X**		
15. Does the country ever face difficulties in obtaining sufficient quantities of measles vaccine? If so, why, and what could be done to alleviate the situation?		**X**		
16. To what extent are measles booster vaccinations being offered and taken up by people who may not have full immunity? What are the challenges in this?		**X**		

17. To what extent was post-exposure prophylaxis for measles available and used during the recent measles epidemic? Were there any under-served areas or groups? Details.		**X**		
**SURVEILLANCE AND CONTACT TRACING**				
18. Do you think that the measles surveillance system functioned well in identifying and tracking the recent epidemic? What gaps, if any, were identified in the system, and how could these be addressed?	**X**			
19. Was contact tracing conducted during the recent measles epidemic? Who was responsible for this, and what challenges, if any, were faced?	**X**			
**SCHOOLS**				
20. Do schools play a role in routine measles vaccination? Details.		**X**	**X**	
21. What was the role of the school authorities during the recent measles epidemic?		**X**		
**HEALTH COMMUNICATION**				
22. To what extent is/was the public aware of and concerned about the recent measles epidemic?	**X**		**X**	**X**
23. What steps were taken to inform the public about the epidemic by the health authorities? Who was responsible for implementing the communications strategy?	**X**			**X**
24. Do you think that the public was satisfied with the information that they received from the government and the media about the measles epidemic? In general, does the public trust the health information they receive? Details.	**X**		**X**	**X**
25. What are the potential challenges of working with the media/authorities [*depending on who is being asked the question*] during a measles epidemic? How can these best be addressed?	**X**			**X**

26. Describe your experience of reporting on the recent measles epidemic. How have your working relations been with the authorities? Did they actively engage you and/or your colleagues in their public information campaigns regarding the epidemic?				**X**
**MEASLES ELIMINATION**				
27. What steps, if any, are being taken in the country towards the elimination of measles, within the context of WHO's European measles elimination plans? What are the challenges, and how are these being addressed? Would you say there is good political commitment to achieving this goal? Details.	**X**			
**CROSS-BORDER COMMUNICATION**				
28. To what extent was there communication with neighbouring, EU, and other countries about the recent measles epidemic, and what was the objective of this communication? Which institutions in which other countries were your country's key points of contact? Were there any challenges in this cross-border communication, or was the process straightforward? Details.	**X**			
**TOTAL NUMBER OF QUESTIONS PER INTERVIEWEE CATEGORY**	**17**	**18**	**5**	**5**

POLIO QUESTIONS FOR EU ASSESSMENTS – EU Member States

POLIO

	National polio coordinator	National immunization programme	Ministry of the Interior/ Migration office	Health journalist
**GENERAL**				
1. According to the RCC's report from June 2015 your country received an intermediate risk score regarding an outbreak of poliovirus disease [*high risk / Romania*]. What is your view on this score?	X			
2. What has been done since then to lower the risk score in time for the next assessment? Which organisations are involved in these activities? Do you think that these actions are sufficient, and what are the main challenges?	X			
3. In broad terms, what is being done in your country to implement the Polio Eradication and Endgame Strategic Plan 2013-2018? Where do you see the biggest challenges in this? Details. Would you say there is good political commitment to implement the strategy? [*to be asked at end of interview*]	X	X		
**PREPAREDNESS**				
4. [*See if we can get a copy of the national (polio-specific) preparedness and response plan and/or the most recent annual report of the National Polio Certification Committee*] Are you satisfied with the national polio preparedness and response plan or do you think that there are there areas that need to be improved? What funding mechanisms are in place to ensure that polio preparedness and response activities are properly supported?	X	X	X	
5. Does the national preparedness and response plan specify activities for regional/local level, or are there sub-national plans?	X	X	X	

POLIO QUESTIONS FOR EU ASSESSMENTS – EU Member States

	National polio coordinator	National immunization programme	Ministry of the Interior/ Migration office	Health journalist
6. Which organisations and societal sectors are involved in the national polio preparedness and response plan? What command and control structures have been put in place to coordinate the various sectors and organisations in their response to a potential polio outbreak?	X			
7. When has the polio preparedness and response plan last been reviewed and/or exercised? What challenges were encountered and consequently, what changes have been implemented, if any? Are you aware of any obstacles to implementing the lessons learned? [*If available, see if we can get a copy of the exercise report*]	X	X		
8. How, if at all, do you think that Europe's polio-free status (since 2002) has affected preparedness planning (and awareness) in your country?	X	X	X	X
**VACCINATION AND HEALTH SYSTEMS ISSUES**				
9. What actions are taken to implement the European Vaccine Action Plan in your country?		X		
10. Are you confident that the health services have sufficient support – clinically, administratively, and managerially – to effectively and safely manage a polio outbreak in the country? What might be still needed, and who would be responsible for providing this?	X			
11. Please describe your polio vaccination program. [*If available, see if we can get a copy of any relevant documentation*.] What is the mechanism by which you ensure that you have access to sufficient IPV for routine vaccination?		X		

	National polio coordinator	National immunization programme	Ministry of the Interior/ Migration office	Health journalist
12. What are the challenges in achieving the 95% coverage rate (for the third dose) required for population immunity? Are there any under-vaccinated / hard-to-reach / lower-uptake groups and if so, which are these? What is done to increase the immunization coverage of those groups? What, if anything, is done to ensure that people entering the country are properly immunized? Details.	X	X	X	
13. Are you satisfied with the accuracy of routine polio immunization coverage data in the country, both for the whole population and for any under-vaccinated groups? Please explain.	X	X		
14. Are you satisfied with the availability of bivalent OPV in case of an outbreak, now that OPV2 has been withdrawn?	X	X		
15. To what extent are polio booster vaccinations offered and taken up by people who are potentially at risk of infection but who may not have full immunity? What are the challenges in this?		X	X	
**RISK COMMUNICATION**				
16. To what extent is the population aware of (and concerned with) the risks connected with polio, and what is the general attitude to polio vaccination?	X	X		X
17. To what extent does vaccine hesitancy exist in the country? Which groups are most resistant to vaccination, and what drives their resistance? What actions are currently taken to promote polio vaccination in these groups? Do you consider this to be sufficient? If not, what more could be done?	X	X		X

POLIO QUESTIONS FOR EU ASSESSMENTS – EU Member States

	National polio coordinator	National immunization programme	Ministry of the Interior/ Migration office	Health journalist
18. Do you have a national strategy in place for communication with the population in case of a polio outbreak?<br>*If yes*: Who is responsible for implementing the strategy, and which communication channels are included (including social media)? What kind of training is provided to official spokespeople? Where can clinicians and media contacts get advice and up-to-date information in the event of a polio outbreak?<br>*If no*: Why not?	X	X		
19. Have health promotion materials been produced for use in the event of a polio outbreak? If not, at what point, and by whom, would this process be activated?	X	X		
20. What would you say are the main challenges of working with the media/authorities [*depending on the interviewee*] in relation to providing information about vaccine-preventable diseases such as polio?		X		X
21. Do you think that the public was satisfied with the information they received from the government/the media during previous health alerts, e.g. Ebola 2014/2015 or H1N1 2009? Does the public trust the health information they receive? Details.		X		X
22. Describe your experience of reporting on previous disease outbreaks. How have your working relations been with the authorities? Have they actively engaged you and/or your colleagues in their public information campaigns regarding the outbreak?				X

POLIO QUESTIONS FOR EU ASSESSMENTS – EU Member States

	National polio coordinator	National immunization programme	Ministry of the Interior/ Migration office	Health journalist
**CROSS BORDER ISSUES**				
23. Do you consider that the three officially sanctioned reporting and notification systems (IHR, EWRS, TESSy) will provide your country with adequate polio-relevant information from other countries, and will work efficiently and effectively in the event of an outbreak. What challenges are there in this for your country, if any?	X			
24. Are you aware of any protocols or relevant contacts between your country's authorities and their counterparts in neighbouring countries with regard to polio preparedness and response? *If yes*, what form has cross-border cooperation taken, who has been involved, what has been the outcome, and what have been the challenges in the process? *If no*, has the possibility of such joint work been discussed?	X	X	X	
**POLIO SURVEILLANCE**				
25. What type of polio surveillance is performed in your country (i.e. AFP, environmental, enterovirus)? Details, challenges (including regarding laboratory resources, training etc.)? Which organisations have defined roles in polio surveillance? Which parts of the population / country are covered by the surveillance system?	X	X	X	
26. Is there currently any sort of enhanced clinical, environmental or enterovirus surveillance ongoing for polio in any specific groups, and if so, how is this work structured? Are there any challenges in this work? Are the underlying population data to guide the surveillance efforts and to calculate coverage accurate and up to date?	X	X		
27. Are you satisfied that the country's surveillance system would detect a polio outbreak, should it occur? Are enough resources allocated for surveillance? Where do you see the main challenges and how could these be addressed?	X	X	X	

	National polio coordinator	National immunization programme	Ministry of the Interior/ Migration office	Health journalist
28. Where are clinical, and (if appropriate) where are environmental polio samples analyzed, in your own laboratory or are you sending samples elsewhere for (routine) analysis? Are there any funding or other challenges for this work? Are you satisfied with the logistics arrangements and security protocols regarding storage and transport of polio samples?	X			
**POLIO CONTAINMENT**				
29. What is being done in your country to implement the Global Action Plan (GAPIII)? What are the plans for destroying all WPV and OPV materials in the country (under GAP III)? How much WPV/OPV material is there thought to be, and how secure is it? What do you think you might not know, i.e. where might there be polio-contaminated samples that are not currently known about?	X	X		
**TOTAL NUMBER OF QUESTIONS**	**23**	**22**	**8**	**6**
